# Antimicrobial and Antioxidant Activities of Natural Compounds

**DOI:** 10.1155/2018/1945179

**Published:** 2018-06-06

**Authors:** Letizia Angiolella, Gianni Sacchetti, Thomas Efferth

**Affiliations:** ^1^Department of Public Health and Infectious Diseases, Sapienza, Università di Roma, Rome, Italy; ^2^Department of Life Sciences and Biotechnology, University of Ferrara, Ferrara, Italy; ^3^Department of Pharmaceutical Biology, Institute of Pharmacy and Biochemistry, Johannes Gutenberg University, Mainz, Germany

Since ancient times, medicinal plants have traditionally been used for the treatment of different diseases. Nowadays, plants are considered a valuable source of unique natural compounds used in the development of antidiabetic, anti-inflammatory, anticancerous, and antimicrobial drugs. Bacteria, fungi, and viruses are responsible for a range of human diseases. Microbial invasion and its virulence can cause damage to the host cells. Effective antimicrobials have been developed over the years; however, a dramatic increase in resistance to antimicrobial drugs has been observed. The resistance to these drugs has also led to the reemergence of old infectious diseases. Oxidative stress is characterized as an imbalance between the production of reactive species and antioxidant defense activity, and its enhanced state has been associated with many of the chronic diseases such as cancer, diabetes, and neurodegenerative and cardiovascular diseases. Many studies have investigated the antioxidant properties of natural products, but, despite the large number of natural products that are currently studied, the search for new natural compounds with antimicrobial and antioxidant activities still remains a growing research area.

Our special issue, which had opened for 6 months in the second half of 2017, focused on highlighting the recent research on new trends and on new antimicrobial and antioxidant natural compounds obtained from medicinal plants. There were both in vitro and in vivo studies with the aim of relating antimicrobial and antioxidant properties of extracts, fractions, synergistic mixtures, and single pure compounds to possible treatments of human disorders and diseases.

W. A. S. S. Weerakoon et al. evaluated Antioxidant Potentials of Sudarshana Powder through in vitro and in vivo approaches. Antioxidant effects were checked in vitro by ABTS assay, while the Lipid Peroxidation (LPO) in serum was used to detect in vivo activities. ABTS assay revealed that the in vitro antioxidant activity of Sudarshana Powder was equivalent to 14.45 *μ*g of standard Trolox® corresponding to an inhibition percentage of the radical formation of 50.93 ± 0.53%. Furthermore, Sudarshana Powder showed in vivo a significant (*p* < 0.01) decrease in the serum level of thiobarbituric acid reactive substance when compared with the control group. The conclusion of the research is that Sudarshana Powder evidenced biological properties in vitro and in vivo that may attest its health value as traditional product.

P. Cheypratub et al. demonstrated the antibacterial activity and mode of actions of* Cyperus rotundus* extract (CRE) against ampicillin-resistant* Staphylococcus aureus* (ARSA) which poses a serious problem for hospitalized patients. The Minimum inhibitory concentrations (MICs) for ampicillin and CRE against all ARSA strains were 64 *μ*g/ml and 0.5 mg/ml, respectively. Checkerboard assay revealed synergistic activity in the combination of ampicillin and CRE at the lowest fractional inhibitory concentration index (FICI) at 0.27. The killing curve assay had confirmed the synergistic and bactericidal activity of the combination against ARSA. This combination caused peptidoglycan and cytoplasmic membrane (CM) damage and an increase in CM permeability of ARSA. So, this CRE proposes the potential to develop a novel adjunct phytopharmaceutical to ampicillin for the remedy of ARSA.

H. O. Elansary et al. described the phenolic profile of the medicinal plants* Asparagus aethiopicus* L.,* Citrullus colocynthis* L.,* Senna alexandrina* L.,* Kalanchoe delagoensis* L.,* Gasteria pillansii* L.,* Cymbopogon citratus*,* Brassica juncea, and Curcuma longa* L. In particular these plants were rich sources of important compounds such as robinin in the fruits and leaves of* A. aethiopicus*; caffeic acid in the tubers of* A. aethiopicus*; quercitrin in the leaves of* G. pillansii*; benzoic acid in the pods of* S. alexandrina*; phenylalanine in the fruit coat and seeds of* C. colocynthis*; hydroxycaffeic acid in the fruits of* C. colocynthis* and roots of* C. longa*; trifoline in the leaves of* K. delagoensis*; rutin in the leaves of* C. citratus*; and esculin in the seeds of* B. juncea*. Further, relatively high antioxidant, antibacterial, and antifungal activities were observed in* C. colocynthis* fruit coat,* S. alexandrina* pods, and* A. aethiopicus* leaves, respectively. Therefore, it was concluded that the fruit coat of* C. colocynthis*, pods of* S. alexandrina*, and leaves of* A. aethiopicus* might be excellent sources of natural products.

B. E. N. Wamba et al. reported the antibacterial activities of methanol extracts of bark and leaves of* Syzygium jambos*, as well as their synergistic effects with selected antibiotics against drug-resistant Gram-positive and Gram-negative bacteria. Extract of the leaves was active against all the 26 strains of* Staphylococcus aureus* and all the 21 strains of* Gram-negative* bacteria tested, within the minimum inhibitory concentration (MIC) range of 32–512 *μ*g/mL. The lowest MIC value of 32 *μ*g/mL was obtained with extract of the leaves against* Staphylococcus aureus* MRSA strain. In Gram-negative bacteria, the lowest MIC value of 64 *μ*g/mL was also obtained against* Enterobacter aerogenes* EA294 and* Klebsiella pneumoniae* K24 strains. Against* S. aureus* strains, antibiotic-modulating activity of extracts at MIC/2 towards more than 70% of the tested strains was obtained when leaves and bark extracts were tested in association with chloramphenicol (CHL). In conclusion, this study demonstrated that* Syzygium jambos* has antibacterial and antibiotic modulating activities.

L. Ma et al. reported the protective effect of allicin against cardiomyocyte apoptosis that was induced by ischemia in vitro and the potential molecular mechanisms that were involved in this antiapoptotic effect. The results indicated that allicin increased H9c2 cell activity and attenuated the rate of apoptosis that was induced by ischemia/hypoxia. Intracellular calcium concentrations significantly decreased in the allicin-treated groups. Bax expression significantly decreased, and Bcl-2 expression increased in allicin-treated rats. Nitric oxide blockade significantly inhibited these effects. Allicin also increased the activity of SOD and NO release and decreased MDA levels. Allicin significantly increased the expression of eNOS, Nrf2, and HO-1 proteins. Collectively, these finding demonstrate that allicin protects H9c2 cells against apoptosis, and this protective effect appears to occur via eNOS/NO pathway-mediated antioxidant activity. Collectively, these findings demonstrate that allicin protects H9c2 cells against apoptosis, and this protective effect appears to occur via eNOS/NO pathway-mediated antioxidant activity.

An interesting paper by J. C. López-Romero et al. shows the seasonal effect on the antioxidant, antiproliferative, and antimicrobial activities of* L. glaucescens *Kunth (LG) leaves extracts. All the LG extracts presented high antioxidant activity and phenolic compounds (PC), with quercitrin and epicatechin being the most abundant. Antioxidant activity and PC were affected by the season; particularly the autumn (ALGE) and summer (SULGE) extracts exhibited the highest values (*p* < 0.05). All extracts presented moderate antiproliferative activity against the cell lines evaluated, with HeLa being the most susceptible of them. However, ALGE and SULGE were the most actives too. About antimicrobial activity, SULGE (MIC 90 < 800 *μ*g/mL; MIC 50 < 400 *μ*g/mL) and SLGE (MIC 50 < 1000 *μ*g/mL) showed a moderate inhibitory effect against* S. aureus*. These findings provide new information about the seasonal effect on the PC and biological properties of LG extracts. Clearly, antioxidant activity was the most important with respect to the other two.

An article by B. Riaz et al. described the toxicity, phytochemical composition, and enzyme inhibitory activities of some 37 indigenous weed plant extracts in fruit fly,* Drosophila melanogaster,* used as model organism. In particular, the research profile was designed to evaluate the toxicity of petroleum extract of some weed plants, as* Azadirachta indica*,* Euphorbia prostrata*,* Parthenium hysterophorus*,* Fumaria indica,* and* Chenopodium murale* against* D. melanogaster*. Among all the results acquired, those regarding* E. prostrata* evidenced high mortality (51.64%) at 30% concentration and more toxicity levels (LC50 27.76; *p* value 0.00) after 72 hours.* A. indica* showed high LC50 value (*p* value 0.15) compared to other weed plants. Interestingly, the combination of* E. prostrata* and* Bti* showed highest mortality (100%; LC50 12.49; *p* value 0.00) after 72 hours. Similarly, the same combination caused maximum reduction in the activity of AChE, AcP, AkP, *α*-carboxyl, and *β*-carboxy enzymes. The phytochemical analysis of plant extracts revealed the presence of flavonoids, saponins, tannins, steroids, cardiac glycosides, alkaloids, anthraquinones, and terpenoids. FTIR analysis of* E. prostrata* showed the presence of phenolic compounds.

The antibacterial, antioxidant activity of ethanolic plant extracts of some Convolvulaceae species and their DART-ToF-MS profiling was instead studied by A. Al-Rifai et al. Since* Convolvulus austroaegyptiacus *(CA) and* Convolvulus pilosellifolius* (CP) are commonly used in the Saudi Arabia folk medicine, the authors investigated the possible biological activities (antibacterial and antioxidant) of their total ethanol extracts and various fractions. Total flavonoid contents of the plants were determined by colorimetric method while total phenols were determined by using Folin-Ciocalteu method. In vitro antibacterial activity was checked against* E. coli*,* P. aeruginosa*, and* B. subtilis*: the lowest MIC value of 0.25 mg/mL was recorded with CP extract against* B. subtilis*. The total antioxidant capacity was evaluated by radical scavenging method showing IC50 values corresponding to 21.81, 17.62, and 3.31 *μ*g/mL for CA, CP, and vitamin C, respectively. Some compounds were tentatively elucidated for both plants by direct analysis in real time-time of flight-mass spectrometry.

An article by S. Abu-Lafi et al. reported the antimicrobial activity of olive mill wastewater (OMWW) extract against Gram-positive and Gram-negative yeast. This extract contained three phenolic compounds as hydroxytyrosol, tyrosol, and oleuropein and it has strong antioxidant activity. The OMWW extract showed also positive activities as antibacterial (Gram-positive and Gram-negative) and antifungal activities and activities against yeast. The addition of OMWW extract to olive oil samples has an effect on the stability of olive oil as reflected by its acid value, peroxide value, K232 and K270, and total phenolic content.

J. Lee et al. studied the marine plant* Ecklonia cava* extract and dieckol as tool to attenuate cellular lipid peroxidation in cultured HaCaT keratinocytes exposed to airborne particulate matter with a diameter of <10 mm (PM10). The choice of* Ecklonia cava* as source of compounds with promising protection effects was due to the fact that the total phenolic content of its extract was the highest among the 50 marine plant extracts examined. After the exposure of cultured HaCaT keratinocytes to PM10, in the absence and presence of* E. cava* extract, cell viability and cellular lipid peroxidation were assessed, together with the effects of eckol and dieckol on cellular lipid peroxidation and cytokine expression. The results achieved for cell cultures without* E. cava* extract evidenced that the exposure to PM10 decreased cell viability and increased lipid peroxidation. However, the cellular lipid peroxidation induced by PM10 was attenuated by* E. cava* extract and its ethyl acetate fraction. In fact, dieckol and eckol attenuated the expression of inflammatory cytokines such as tumor necrosis factor-*α* (TNF-*α*), interleukin-1*β* (IL-1*β*), IL-6, and IL-8 in human epidermal keratinocytes stimulated with PM10. However, dieckol was more effective than eckol in attenuating cellular lipid peroxidation in both HaCaT cells and human epidermal keratinocytes. The data collected and discussed in this paper suggested that* E. cava* extract and its constituent dieckol attenuated the oxidative and inflammatory reactions in skin cells exposed to airborne particulate matter.

K. Díaz et al. discussed the isolation and identification of compounds from bioactive extracts of* Taraxacum officinale* (Dandelion) from the southern Chile as a potential source of antibacterial agents, with the aim of identifying novel natural agents with in vitro antibacterial activity against* Staphylococcus aureus*,* Escherichia coli,* and* Klebsiella pneumoniae* strains. The chemical characterizations concerned the identification of compounds present in hexane (Hex) and ethyl acetate (AcOEt) extracts through gas chromatography-mass spectrometry (GC-MS) and nuclear magnetic resonance (NMR). Different sesquiterpene lactones (*α*-santonin, glabellin, arborescin, and estafiatin), a monoterpene (9,10-dimethyltricycle [4.2.1.1  (2,5)] decane-9,10-diol), a phytosterol (stigmasta-5,22-dien-3*β*-ol acetate), terpenes (lupeol acetate, pregn-5-en-20-one-3*β*-acetyloxy-17-hydroxy, and 2-hydroxy-4-methoxy benzaldehyde), and a coumarin (benzofuranone 5,6,7,7-a-tetraaldehyde-4,4,7a-trimethyl) were mainly detected. The results obtained show that the Hex extract was highly active against* Staphylococcus aureus* showing a MIC of 200 *μ*g/mL and moderately active against* Escherichia coli* and* Klebsiella pneumoniae* with MIC values of 400 *μ*g/mL and 800 *μ*g/mL for the other Gram-negative strains tested with* Proteus mirabilis*. The results revealed Dandelion extracts as promising source of biomolecules for possible industrial application in the field of antimicrobial products.

An article by T. Appiah et al. reported the antimicrobial activity of methanol extracts of* Trametes gibbosa*,* Trametes elegans*,* Schizophyllum commune,* and* Volvariella volvacea *against selected microorganisms as Gram-positive, Gram-negative and fungi. The methanol extracts of these mushrooms were found to contain secondary metabolites such as tannins, flavonoids, triterpenoids, glycosides, and alkaloids.* T. gibbosa* and* T. elegans* extracts had antimicrobial activity against test organisms with minimum inhibitory concentration (MIC) ranging from 4 to 20 mg/mL and 6 to 30 mg/mL, respectively, while* S. commune* and* V. volvacea* extracts had MIC range of 6 to 20 mg/mL each. All four mushroom extracts were able to inhibit the growth of Gram-negative and Gram-positive bacteria and their activity was bacteriostatic as confirmed by time killing. Methanol extracts of* T. gibbosa*,* T. elegans*,* S. commune,* and* V. volvacea* exhibited antimicrobial activity and may contain bioactive compounds which may serve as potential antibacterial and antifungal agents.

